# Cardiac lipoma in the interventricular septum: a case report

**DOI:** 10.1186/s13019-015-0275-0

**Published:** 2015-05-09

**Authors:** Dan Li, Weitie Wang, Zhicheng Zhu, Yuefeng Wang, Rihao Xu, Kexiang Liu

**Affiliations:** 1 Department of Cardiovascular Surgery, 2nd Hospital of Bethune, Jilin University, Changchun, Jilin China; 2 Department of Cardiovascular Surgery, Daqing Oilfield General Hospital, Daqing, Heilongjiang China

**Keywords:** Cardiac tumors, Lipoma, Cardiac surgery, Pathology

## Abstract

Primary cardiac tumors are rare. The cardiac lipoma cases have been sparsely reported. We report a case of interventricular septal lipoma complicated with mild tricuspid regurgitation in a 65-year-old Chinese male. The patient presented with shortness of breath after exertion. His diagnosis was made with echocardiogram, cardiac magnetic resonance imaging, cardiac computed tomography and post-operative histopathology. The patient underwent tumor resection and postoperative recovery was uneventful. He was asymptomatic with no recurrence at 8-month follow-up.

## Background

Primary cardiac tumors are uncommon. It has been reported that approximately 75 % of the primary cardiac tumors are benign [1]. Cardiac lipomas account for 8.4 % of the primary tumors of the heart and pericardium [2]. Cardiac lipomas are benign nonmyxomatous neoplasms of the heart, they can occur at any age with an equal frequency in both genders [3] and in almost any locations in the heart. They are not easily clinically diagnosed, and differ in regard to clinical manifestations, locations, morphology, sizes and radiological findings [4]. They are uncommon and usually found in the right atrium and left ventricle. Since cardiac lipomas are well-encapsulated tumors and typically composed of mature fat cells, they are true neoplasms, unlike hypertrophic lipomatosis, in which there is deposition of non-encapsulated mature and fetal adipose tissue [5]. A cardiac tumor in the IVS occurs in only 2 % of cases [6]. Therefore, the combination of a rare tumor (lipoma) in a rare location (IVS) is particularly unusual [7], it is to say, lipoma in the IVS is very rare. Albers was the first to report cardiac lipoma in 1856, and about 60 cases had been reported until 1995 [8]. We present a rare case of lipoma located in the interventricular septum (IVS). The tumor was successfully resected surgically.

## Case presentation

**Fig. 1:**
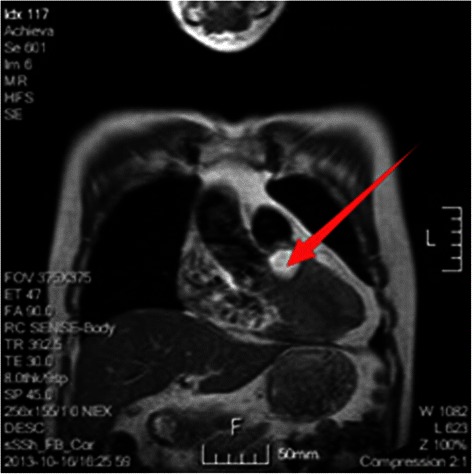
Cardiac MRI showed a round-like signal, measuring 36 × 20 mm, in the upper portion of IVS, anterior to the right coronary sinus of aorta

**Fig. 2:**
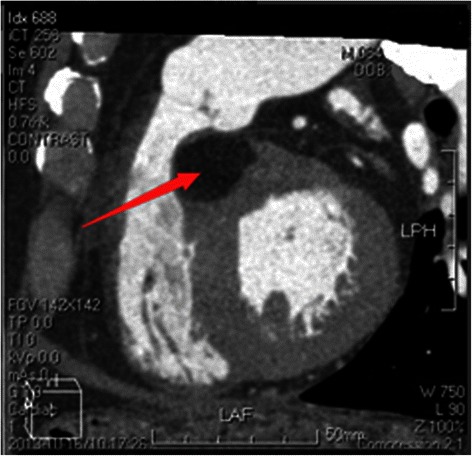
Cardiac CT images with contrast confirmed the existence of a round-like hypodense mass (−117Hu), measuring 37 × 21 mm in its largest diameter, superior to the IVS and anterior to the right coronary sinus of aorta.

A 65-year-old Chinese male experienced chest congestion and shortness of breath after exertion for 11 years, but he had ignored them. He presented with worsening dyspnea and increasing fatigue for the past 3 months. His blood pressure was 133/72 mmHg and his heart rate was 110 beats per min. Heart auscultation revealed grade II ejection systolic murmur at the left parasternal border. Electrocardiogram showed normal sinus rhythm. Transthoracic echocardiogram (TTE) showed a homogeneous parenchymal mass of 27 × 12 mm in size with peripheral hyperechogenicity. The mass located in the mid-basal portion of IVS, anterior to the right coronary sinus. There was mild tricuspid regurgitation (TR) without annular dilatation and cusp prolapse on TTE. Cardiac magnetic resonance imaging (MRI) showed a round-like signal, measuring 36 × 20 mm, in the upper portion of IVS, anterior to the right coronary sinus of aorta. The images of tissue characterization suggested that the mass was consisted of adipose tissue (Fig. 1). Cardiac computed tomography (CT) scan with intravenous contrast confirmed the existence of a round-like hypodense mass (−117Hu), measuring 37 × 21 mm in its largest diameter, superior to the IVS and anterior to the right coronary sinus of aorta (Fig. 2).

**Fig. 3:**
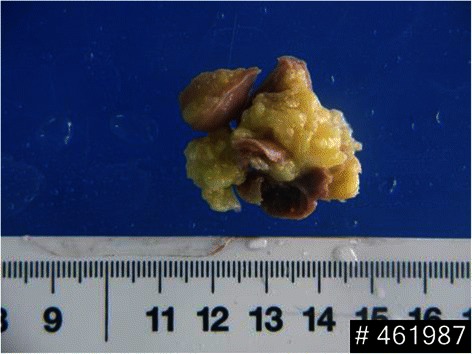
Intraoperative photograph of the resected lipoma

The traditional surgical treatment has been performed. A standard median longitudinal sternotomy was carried out. Cardiopulmonary bypass (CPB) was routinely applied, and myocardial protection through the aortic root infusion of cold cardioplegic solution and topical hypothermia of the heart. Antegrade cardioplegic arrest was achieved after application of an aortic cross-clamp. After longitudinal incision of right atrium and the main pulmonary artery, a yellowish neoplasm was found embedded in the upper portion of IVS beneath the pulmonary valve, projecting into the right ventricular outflow tract. The muscular septum was incised longitudinally and the tumor was excised en bloc from the IVS. The muscular septum and the right ventriculotomy were re-approximated with running 4-0 prolene sutures. Postoperative pathology reported lipoma (Fig. 3), measuring 40 × 30 × 20 mm, encapsulated and composed of mature adipose tissue. The patient recovered well without any complications. He was discharged on the postoperative day 13. His pre-operative symptoms resolved and no further medication treatment is needed. The patient was doing well in his 8 months post operative follow up appointment.

## Conclusions

The clinical manifestations of cardiac lipoma depend on the location of the tumor, and may vary from valvular dysfunction to obstruction of a cardiac chamber, impeding the filling and emptying processes [9]. Symptoms may include heart failure, arrhythmia and syncope. Tumors of the right atrium, interatrial septum, and right ventricle can predispose to arrhythmias [10]. Türkoglu H and his colleagues [11] reported a case of a 24-year-old woman who presented with palpitations and was found to have a lipoma attached to the left side of the IVS. Our patient felt chest congestion and shortness of breath after physical activity for a long time and worsening dyspnea and increasing fatigue whose lipoma located in the upper portion of IVS projecting into the right ventricular outflow tract. The increasing size of the tumor caused underfilling of the right ventricle with rapid deterioration of symptoms. The location and size of the tumor may contribute to the patient’s symptoms. The cardiac lipoma can be diagnosed by echocardiogram, CT and MRI. Echocardiogram is useful to diagnose both benign and malignant tumors. The use of CT scan and MRI are facilitated to the diagnosis of the lipomas and exactly predict the intramyocardial extent and the relationship to other cardiac structures [12]. MRI is particularly useful because it allows the characterization of the tissue [13]. In our case, a mass in the heart was initially detected with transthoracic echocardiogram. CT scan and MRI were further applied to characterize the mass. Post operative pathology confirmed the pre-operative diagnosis of lipoma. So we think that echocardiogram, CT and MRI are necessary to the diagnosis of the cardiac tumor.

Because of potential lethality, theoretically, all cardiac tumors should be indicated for surgery, independing from symptomatology. If there are no contraindications, surgical resection is the treatment of choice for all patients with cardiac neoplasms. The approaches to resect the tumor depends on tumor sizes and locations. In this case, the patient was symptomatic and deteriorated progressively. We recommended surgical treatment and advise this patient to reexamination twice a year after successful treatment.

## Consent

Written informed consent was obtained from the patient for publication of this Case report and any accompanying images. A copy of the written consent is available for review by the Editor-in-Chief of this journal.
